# The Influence of Pulsed Superimposed DC Electric Field Synergistically Inducing Orientation Arrangement of BNNSs on Thermal Properties of Epoxy Composites

**DOI:** 10.3390/mi16101126

**Published:** 2025-09-30

**Authors:** Xiaopeng Wang, Songyuan Li, Zhen Yin, Qi Zhang, Lei Deng, Yiqin Peng, Yan Mi

**Affiliations:** 1 State Grid Tianjin High Voltage Company, Tianjin 300232, China; wxp339@163.com (X.W.); yz963424@126.com (Z.Y.); 2 State Grid Tianjin Electric Power Company, Tianjin 300384, China; lisongyuan1987@163.com (S.L.); zhangqi820323@126.com (Q.Z.); 3 State Key Laboratory of Power Transmission Equipment Technology, School of Electrical Engineering, Chongqing University, Chongqing 400044, China; dl@stu.cqu.edu.cn (L.D.); pyq@stu.cqu.edu.cn (Y.P.)

**Keywords:** epoxy composites, electric field orientation, pulsed superimposed direct current electric field, thermal conductivity

## Abstract

Modern power systems require better heat dissipation and thermal stability, but traditional low-filler composites cannot significantly enhance thermal conductivity. To address this issue, electric field induction technology orientation can efficiently orient boron nitride nanosheets (BNNSs), thereby improving the thermal conductivity of epoxy composites composed of BNNSs as the thermally conductive filler. In this study, an innovative approach employing a pulsed superimposed direct current (DC) electric field to synergistically induce filler orientation is used to construct efficient thermally conductive channels. The study found that the thermal conductivity of the composite prepared by superimposing an 8 kV/mm pulsed electric field on a 30 V/mm DC electric field is about 0.474 W/(m·K), which is 34.66% higher than that prepared by only a pulsed-induced field and 17.5% higher than the theoretical superposition value. Similarly, the composite prepared by superimposing a 4 kV/mm pulsed electric field on a 70 V/mm DC electric field increased to about 0.464 W/(m·K), which is 27.47% higher than that prepared by only a DC-induced field and 12.4% higher than the theoretical superposition value. These results indicate that the superimposed electric field treatment synergistically improves the thermal conductivity of the composite. Compared to other materials, composites prepared using the superimposed pulsed and DC electric field induction also exhibit superior thermal stability. This strategy effectively addresses the issue of material thermal aging caused by insufficient thermal conductivity, providing innovative ideas and a solid theoretical foundation for material design and thermal management.

## 1. Introduction

In recent years, the economy has developed rapidly and electricity demand has grown, so power system equipment is moving towards higher power. This has caused the heat generated per unit volume in power devices to increase sharply. Epoxy resin (EP) is widely used as an insulating material in power systems because it has good corrosion resistance, electrical insulation, and adhesion. But when power equipment runs at high temperatures for a long time, EP-based insulation materials age faster, which finally shortens the equipment’s service life [1,2]. Enhancing the thermal conductivity of insulating materials through various approaches can effectively improve their heat dissipation performance. This not only helps prevent equipment from overheating and premature aging, but also contributes to extending the service life of the equipment to a certain extent [3,4].

A commonly used approach to enhance the thermal conductivity of polymer materials is to incorporate high-thermal-conductivity fillers into the polymer matrix to fabricate polymer-based composites [5,6]. At high filler loadings, the electrical and mechanical properties of polymer composites tend to deteriorate. Conversely, at low filler contents, the fillers are often dispersed in isolation within the polymer matrix, forming “islands”, which significantly limits the enhancement of thermal conductivity. Therefore, developing thermally conductive insulating polymer composites with low filler loading remains both a current research hotspot and a major challenge. Studies have shown that reorienting a small amount (volume fraction < 20%) of thermally conductive fillers to form interconnected or bridged pathways within the polymer matrix is an effective strategy to improve thermal conductivity [7,8,9,10].

Common techniques include the hot-pressing method [11,12], which achieves a high degree of filler orientation under elevated temperature and pressure to construct thermal conduction pathways. However, it is prone to forming orientation gradients in thick-section materials, leading to non-uniform properties. The ice-templating method [13,14] is widely employed in biomaterials due to its environmentally friendly nature and easy processing, yet challenges regarding uniformity and efficiency still need to be addressed. The stretch-induced alignment techniques [15,16,17] can effectively enhance the thermal conductivity of flexible films through polymer stretching. Magnetic field induction [18,19,20] applies external magnetic fields to orient magnetically anisotropic fillers within a polymer matrix, though this technique is costly and necessitates fillers with inherent magnetic properties. Electric field induction [21,22,23] polarizes and aligns fillers within polymer prepolymers, thus creating thermal conduction chains along the direction of the applied electric field. It is considered one of the most efficient and direct methods to orient fillers within the polymer matrix and form a thermally conductive network.

Over the past decade, the electric field orientation has been extensively studied by many researchers [24,25,26]. Mi et al. [27] applied microsecond pulsed electric fields to align BNNSs in epoxy composites. At 10 wt% filler loading, composites processed under a 12 kV/mm, 100 Hz pulsed field showed a thermal conductivity of 0.588 W/(m·K), exceeding a 200% enhancement compared to pure EP. Yang et al. [28] used a nanosecond pulsed electric field of 0–120 V/mm to induce the orientation of BNNSs for preparing composites with high energy storage performance. Bi et al. [29] utilized a direct current electric field to orient BN in epoxy resin for fabricating oriented composites. The thermal conductivity of the oriented composites was 1.6 times higher than that of the random BN/EP composites. Sima et al. [30] employed a combination of magnetic orientation and the ice-templating method to fabricate cactus-like dual-oriented magnetic SiC and BN networks in epoxy composites. At a filler volume fraction of 20 vol%, the thermal conductivity reached 3.35 W/(m·K). Wang et al. [31] used BN and SiC as fillers and adopted solution blending, electrospinning, and hot pressing to prepare high-thermal-conductivity poly (ether ketone nitrile) (PEK-CN) composites. Their results showed that with 20 wt% BN and 10 wt% SiC, the thermal conductivity increased from 0.251 W/(m·K) (pure PEK-CN) to 0.812 W/(m·K), and the glass transition temperature (Tg) increased from 228.1 °C to 232.4 °C, indicating enhanced thermal stability.

Previous work by our group found that pulsed electric fields enhance the thermal conductivity of composites mainly by improving the local alignment of BN nanosheets (thermal conductivity of 0.352 W/(m·K) at 8 kV/mm), while DC electric fields enhance the global alignment of BN nanosheets, leading to a thermal conductivity of 0.364 W/(m·K) at 70 V/mm [32]. In summary and inspired by this, this study takes epoxy resin as the research object and BNNSs as the filler. The pulsed superimposed DC electric field is used to induce the oriented arrangement of BNNSs, form an efficient thermal conductive network, and prepare low-filled high thermal conductive composites. The correlation between the degree of BNNS orientation induced by the superimposed electric field and the thermal conductivity of the composites is explored. Thermal stability tests are also conducted on representative composites to evaluate the thermal properties of the superimposed induced composites.

## 2. Materials and Methods

### 2.1. Materials

This research employed E-51 epoxy resin, along with methyl tetrahydrophthalic anhydride (MTHPA) as the curing agent and DMP-30 as the accelerator. These materials were sourced from Huakai Resin Co., Ltd., located in Chongqing, China. The filler utilized consisted of boron nitride nanosheets (BNNSs), which had an approximate thickness of 50 nm and diameters spanning from 0.5 to 5 μm. These BNNSs were provided by Beijing Deke Daojin Science and Technology Co., Ltd., also based in Beijing, China. No surface treatment was applied to the BNNSs.

### 2.2. Sample Preparation Method

First, the epoxy resin was mixed with 99.9% boron nitride nanosheets (BNNSs), with a BNNS loading of 10 wt% and a volume fraction of approximately 5.6% in this study. The resulting combination was then subjected to magnetic stirring for a duration of 1 h, and this is followed by 30 min of probe-type ultrasonic treatment. Subsequently, 8.5 g of a curing agent and 0.2 g of an accelerator are incorporated into the mixture, which is then ultrasonicated once more for 30 min. After that, the mixture, which was dispersed through ultrasonication, was degassed under vacuum for 30 min. The suspension obtained from this process was then poured into a polytetrafluoroethylene (PTFE) mold. The curing process was executed in two phases: the first phase lasted for 2 h at a temperature of 90 degrees, and the second phase also lasted for 2 h but at 110 degrees. In the initial phase of curing, the epoxy prepolymer kept a low viscosity, which helps in the alignment of BNNSs. For this reason, an electric field was applied during the first 40 min of the curing process. The preparation process of the relevant samples and the schematic diagram of the electric field treatment procedure have been detailed in our previous studies [32].

### 2.3. Induction Power Supply

**Figure 1:**
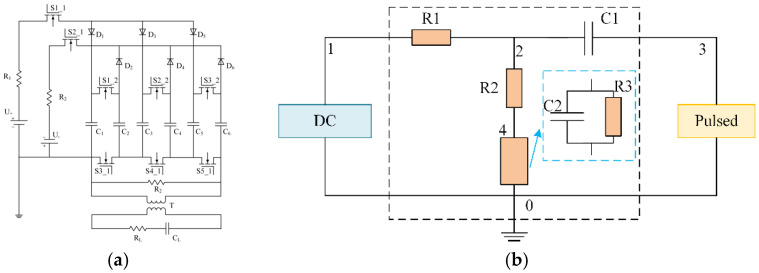
Pulse generator topology and combined coupling circuit diagram. (**a**) Pulse generator topology; (**b**) combined coupling circuit.

This study utilized a pulsed power supply, a direct current (DC) power supply, and a combined power supply to generate the electric fields required for inducing the orientation of BNNSs. The pulsed power supply and combined power supply were custom-made by the research team, as shown in Figure 1. The electrode shape is a square plate with dimensions of 17 mm × 17 mm, and the spacing between the electrode plates is 1.7 mm.

The pulse generator is based on the Marx principle, as illustrated in Figure 1a. The electrodes of the prepolymer mold are connected to the secondary side of the pulse transformer, where CL represents the equivalent capacitance of the prepolymer sample, and RL is the protection resistor. The circuit diagram of the combined coupling circuit is shown in Figure 1b. Its function is to superimpose the DC voltage and the pulsed voltage across the load. In the combined coupling circuit, R1 is the protection resistor for the DC power supply, C1 is the DC blocking capacitor, R2 is the protection resistor for the pulsed power supply, and the load is the epoxy resin prepolymer, which is equivalent to an RC parallel circuit composed of R3 and C2. Due to the small spacing between the electrode plates and the resulting good uniformity, no discharge was observed during the actual experiments. Additionally, the waveform was monitored in real-time using an oscilloscope, confirming that the waveform remained stable and the electric field exhibited excellent stability throughout the experiments.

### 2.4. Material Performance Testing

Microstructural observations of sample surfaces and cross-sections were conducted via scanning electron microscopy (SEM, Nova400, FEI, Hillsboro, OR, USA). X-ray diffraction (XRD, PANalytical X’Pert Powder, Spectris, Almelo, The Netherlands) characterized BNNS alignment in epoxy. Through-thickness thermal conductivity was measured using a laser flash apparatus (LAF467HT, Netzsch, Bavaria, Germany), with the measurement direction kept parallel to the applied electric field direction. Thermogravimetric analysis (TGA 2, Mettler Toledo, Columbus, OH, USA) evaluated thermal stability, while differential scanning calorimetry (DSC3+, Mettler Toledo) determined glass transition temperatures. Dynamic mechanical analysis (DMA Q850, TA, Boston, MA, USA) measured glass transition temperatures and storage moduli.

## 3. Results and Analysis

### 3.1. Cross-Sectional SEM Images

**Figure 2:**
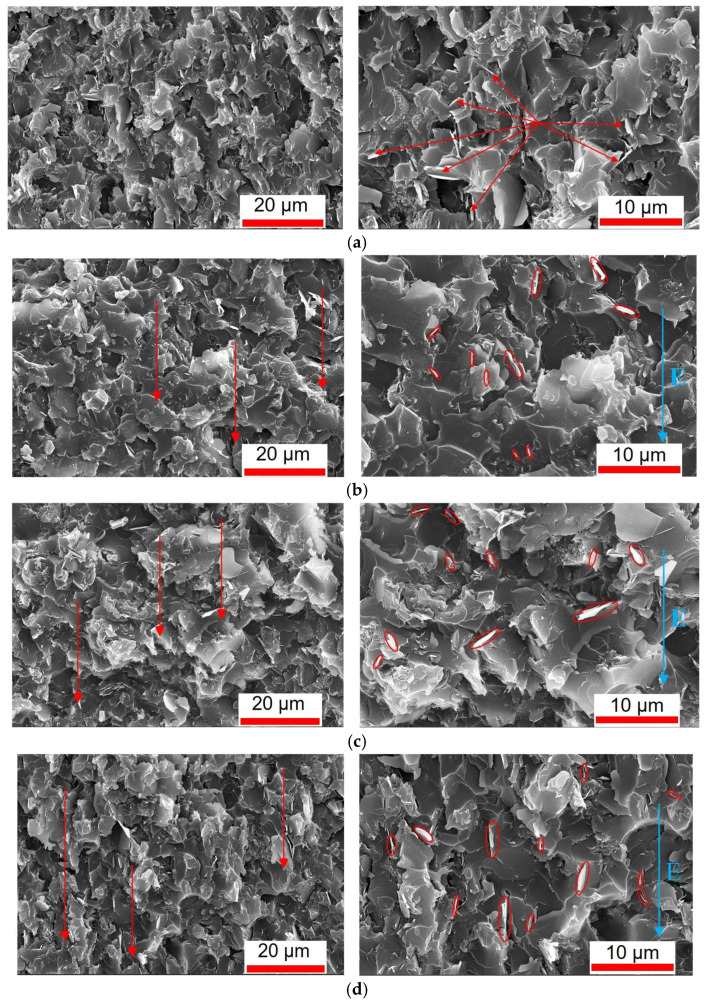
SEM Images of different samples. (**a**) Random sample; (**b**) pulse-induced sample; (**c**) DC-induced sample; (**d**) combined-induced sample.

The cross-sections of the randomly structured sample, the pulse-induced sample (8 kV/mm), the DC-induced sample (70 V/mm), and the combined-induced sample (8 kV/mm + 70 V/mm) were observed using scanning electron microscopy. Figure 2 presents the cross-sectional morphology images of each sample (10 wt% BNNS), with the direction of the applied electric field indicated in the images.

As shown in Figure 2a, BNNSs in the randomly structured sample are dispersed randomly within the epoxy matrix. There is no apparent formation of chain-like structures between the BNNSs, and their orientation is disordered, with nanosheets distributed in various directions. In contrast, for the pulse-induced sample, as shown in Figure 2b, a certain degree of orientation can be observed, as indicated by the red arrows. Many BNNSs are aligned parallel to the electric field direction, indicating that under the induction of an 8 kV/mm pulsed electric field, the BNNSs reoriented toward the field direction and moved closer to each other, forming partial chain-like structures. For the DC-induced sample, as shown in Figure 2c [32], a similar directional tendency is present. However, only a small number of BNNSs are aligned parallel to the electric field, suggesting that although the 70 V/mm DC electric field caused the BNNSs to move closer and form chain-like structures, the relatively low field strength was insufficient to induce significant rotation of the BNNSs. In the case of the combined-induced sample, shown in Figure 2d, the BNNSs exhibit not only clear orientation but also a greater number of nanosheets aligned parallel to the electric field. This indicates that under the combined induction of an 8 kV/mm pulse and a 70 V/mm DC field, the BNNSs experienced both rotational alignment and mutual attraction, leading to the formation of pronounced chain-like structures.

Comparing the SEM results of the four samples—random, DC-induced, pulse-induced, and combined-induced—it is evident that all electric field-assisted processes promoted a certain degree of directional alignment among the BNNSs within the epoxy matrix. In addition, the relevant SEM-EDS analysis has been placed in the Supplementary Material and Figure S1.

### 3.2. XRD Patterns of Nanocomposites and Average Orientation Angle of BNNSs

Since SEM images of the composites can only reflect the local orientation of BN nanosheets, XRD analysis is required to quantitatively evaluate the average orientation angle of BNNSs within the composites.

**Figure 3:**
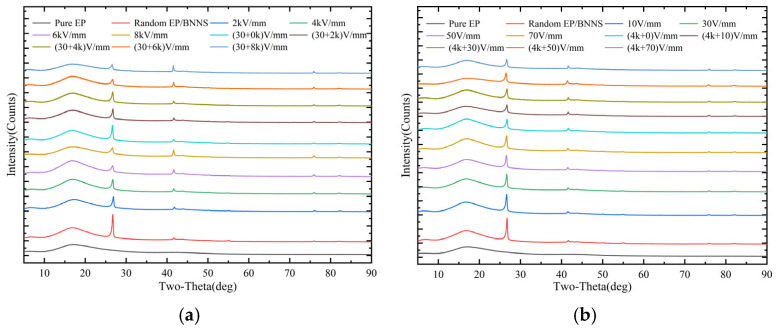
XRD patterns of composites. (**a**) DC-pulsed fields (30 + X kV/mm); (**b**) pulsed-DC fields (4 kV/mm + X).

The XRD patterns of different composites are shown in Figure 3. Compared with the diffraction pattern of pure epoxy resin, the BNNS/EP composites exhibit several additional peaks in the range of 5–90°, with prominent peaks at 26.76° and 41.60°, which correspond to the characteristic diffraction angles of BNNSs. This indicates that the new diffraction peaks originate from the incorporation of BNNSs.

The XRD patterns provide information on the diffraction peaks of BNNSs at various angles in the 5–90° range. To more comprehensively evaluate the orientation of BNNSs, the average orientation angle $\bar{\varphi }$ can be calculated from the XRD patterns using Equation (1), allowing for a quantitative assessment of the BNNS alignment [33]:

(1) $$\bar{\varphi }=\frac{\sum \left(I_{hkl}\times \phi_{hkl}\right)}{\sum I_{hkl}}$$

In the equation, *I_hkl_* represents the intensity of the diffraction peak, and *hkl* is the orientation angle, defined as the angle between the crystal plane (*hkl*) and the base plane (00L). The orientation angle can be calculated using Equation (2) [33]:

(2) $$\cos\phi_{hkl}=\frac{\frac{\sqrt{3}}{2c}l}{\sqrt{h^{2}+k^{2}+hk+\frac{3a^{2}}{4c^{2}}l^{2}}}$$

In the equation, *a* and *c* are the crystal lattice parameters of boron nitride: *a* = 0.2173 and *c* = 0.6657.

**Figure 4:**
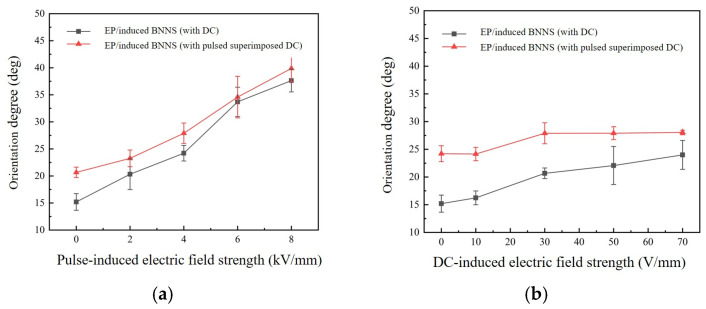
Average orientation angles of composites. (**a**) DC-pulsed fields (30 + X kV/mm); (**b**) pulsed-DC fields (4 kV/mm + X).

Figure 3 presents the XRD diffraction peak results for composites induced by single electric fields and combined electric fields. Specifically, Figure 3a shows the XRD patterns of composites induced by a single pulsed field and combined DC-pulsed fields (30 + X kV/mm), while Figure 3b shows the XRD patterns of composites induced by a single DC field and combined pulsed-DC fields (4 kV/mm + X). The XRD data were processed, and the average orientation angles of BNNSs in different composites were calculated using Equations (1) and (2). The results are shown in Figure 4. Figure 4a presents the average orientation angles of composites induced by a single pulsed field and by combined DC-pulsed fields (30 + X kV/mm). Figure 4b shows the average orientation angles of composites induced by a single DC field and by combined pulsed-DC fields (4 kV/mm + X).

From Figure 4a, it can be seen that the average orientation angles of both the single pulsed field-induced composites and the combined DC-pulsed field (30 + X kV/mm) composites increase with the rising pulsed field strength, indicating that the increase in pulsed field strength promotes the reorientation of the BNNSs. However, the average orientation angle of the combined DC-pulsed field composites does not show significant improvement compared to that of the single pulsed field composites, suggesting that the addition of the DC field does not further enhance BNNS rotation.

### 3.3. Thermal Conductivity of the Composite

Thermal conductivity tests were conducted on various composites, each with a size of 10 × 10 × 0.5 mm^3^, and the results were compiled. Laser flash apparatus parameters: laser voltage 250 V, pulse width 0.6 ms, analysis model: standard model. Thermal conductivity is given by Equation (3):

(3) $$\lambda =\alpha \;*\;\rho \;*\;C_{p}$$

*λ* is the thermal conductivity, with units of W/(m·K), α is the thermal diffusivity, with units of m^2^/s, *ρ* is the material density, with units of kg/m^3^, and *C_p_* is the specific heat capacity of the material, with units of J/(kg·K).

**Figure 5:**
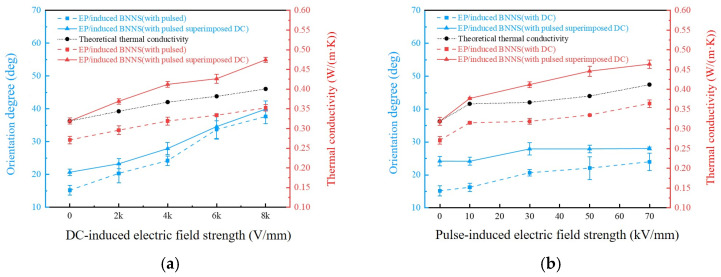
Thermal conductivity of composites. (**a**) DC-pulsed fields (30 + X kV/mm); (**b**) pulsed-DC fields (4 kV/mm + X).

Combined with the average orientation angles of the composites, Figure 5 compares the thermal conductivity of the composites. Figure 5a shows the thermal conductivity of composites induced by a single pulsed field and by combined DC-pulsed fields (30 + X kV/mm). Figure 5b shows the thermal conductivity of composites induced by a single DC field and by combined pulsed-DC fields (4 kV/mm + X).

To quantitatively analyze the relationship between the thermal conductivity of single-field induced composites and superimposed-field induced composites, this study examines whether the combination of pulsed and DC electric fields can produce a synergistic effect in enhancing the thermal conductivity of the composites. The theoretical thermal conductivity of the superimposed-field induced composites under the corresponding parameters is calculated using Equation (4):

(4a) $$\lambda_{P}=\lambda_{R}+\Delta \lambda_{P}$$

(4b) $$\lambda_{D}=\lambda_{R}+\Delta \lambda_{D}$$

(4c) $$\lambda_{PD}=\lambda_{R}+\Delta \lambda_{D}+\Delta \lambda_{P}$$

In the equation, *λ_P_* represents the thermal conductivity of the pulsed induction sample; λ*_R_* represents the thermal conductivity of the random sample; Δ*λ_P_* represents the increase in thermal conductivity of the pulsed induction sample compared to the random sample; *λ_D_* represents the thermal conductivity of the DC induction sample; Δ*λ_D_* represents the increase in thermal conductivity of the DC induction sample compared to the random sample; and *λ_PD_* represents the theoretical thermal conductivity of the superimposed induction sample. According to Equation (4), the thermal conductivity of the superimposed induction composites is integrated into Figure 5.

As shown in Figure 5, the thermal conductivity of the composite prepared by the superimposed induction field is higher than that of the composite prepared by a single induction field. Moreover, the thermal conductivity of the superimposed induction composite also exceeds the theoretical thermal conductivity, indicating that under the action of the superimposed electric field, the thermal conductivity of the composite is synergistically enhanced. This reflects the synergistic effect of the pulsed superimposed DC electric field on the thermal conductivity of the composite.

Specifically, the sample prepared under the superimposed induction field of 30 V/mm DC and 8 kV/mm pulse shows an increase in thermal conductivity to about 0.474 W/(m·K), which is a 74.91% improvement compared to the random sample (0.271 W/(m·K)), a 148.2% increase compared to pure epoxy resin, and 17.5% higher than the theoretical superposition value. The sample prepared under the superimposed induction field of 4 kV/mm pulse and 70 V/mm DC exhibits a thermal conductivity of approximately 0.464 W/(m·K), representing a 71.22% increase over the random sample, a 142.9% increase relative to pure epoxy resin and 12.4% higher than the theoretical superposition value. Table 1 presents a comparison of the thermal conductivity of BNNSs or other fillers under different electric field techniques. It can be seen that the pulsed superimposed DC electric field technology offers a notable improvement in thermal conductivity [21,22,34,35,36].

In addition, we used the Maxwell model [37,38] to calculate the theoretical thermal conductivity of composite materials before applying an external electric field, as expressed in Equation (5):

(5) $$\lambda_{c}=\lambda_{1}*\frac{\lambda_{2}+2\;*\;\lambda_{1}-2\;*\;v_{2}\;*\left(\lambda_{1}-\lambda_{2}\right)}{\lambda_{2}+2\;*\;\lambda_{1}+v_{2}\;*\left(\lambda_{1}-\lambda_{2}\right)}$$

where $\lambda_{c}$ is the thermal conductivity of composite material with units of W/(m·K), $\lambda_{1}$ = 0.19 is the thermal conductivity of the matrix with units of W/(m·K), $\lambda_{2}$ = 360 is the thermal conductivity of the filler with units of W/(m·K). $v_{1}$ is the volume fraction of the matrix, $v_{2}$ is the volume fraction of the filler. Considering the random distribution and flaky characteristics of BNNSs, the theoretical thermal conductivity is 0.224 W/(m·K). By comparing the theoretical and experimental values, it can be seen that the application of a pulsed superimposed DC electric field can effectively enhance the thermal conductivity of the composite.

**Figure 6:**
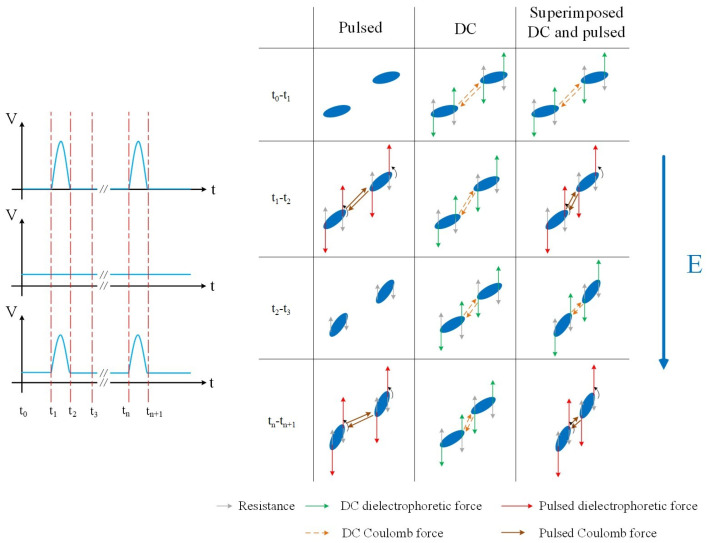
Force and motion state of BNNSs under different electric fields.

The movement process of BNNSs under the pulsed superimposed DC electric field is analyzed as follows. Figure 6 illustrates that under different electric fields, the filler is subjected to dielectrophoretic force, viscous drag, and Brownian motion. Under the influence of the electric field, BNNSs tend to align along the field direction. However, the high viscosity of the EP creates resistance and hinders the BNNSs from moving and orienting quickly. Under the pulsed electric field, BNNSs can overcome this resistance, reorient along the field direction, and approach each other to form partial chain-like structures [31]. Under the DC electric field, BNNSs also tend to align, but the relatively low field strength is not enough to cause significant rotation. In addition, BNNSs also exhibit random Brownian motion within the EP, which interferes with their orientation process induced by the electric field. This interference becomes more pronounced when the nanosheets are smaller in size and at higher temperatures. Only when the dielectrophoretic force exceeds the resisting forces can the filler reorient and move [39,40]. Moreover, BNNS particles that are close to each other can overcome these resistances and approach one another due to Coulombic attraction [41,42]. In Figure 6, the resisting force refers to the resultant ideal force of viscous drag and thermal motion resistance opposing reorientation.

During the process of inducing the orientation of boron nitride nanosheets (BNNSs) using an electric field, distinct behaviors are observed at different stages. In the initial stage (t_0_–t_1_), the pulsed electric field, being at its low level, exerts no significant effect, and BNNSs remain unaligned. At this time, the low field strength of the DC and superimposed electric fields primarily drives the BNNSs to approach each other under Coulombic forces. As the field strength increases (t_1_–t_2_), the pulsed electric field generates substantial dielectrophoretic and Coulombic forces, which overcome resistance to achieve partial reorientation of BNNSs and reduce inter-sheet distances. Meanwhile, the DC field continues to mainly promote particle proximity without significantly affecting alignment. In contrast, the superimposed electric field, under the combined high-field conditions, significantly enhances both dielectrophoretic and Coulombic forces based on the initial minor alignment, thereby strongly facilitating BNNS rotation and further reducing their spacing. In the subsequent low- or zero-field stage (t_2_–t_3_), BNNS motion under the pulsed field slows down due to resistance, while the DC and superimposed fields continue to promote the global arrangement of BNNSs. Finally (t_n_–t_n_₊_1_), a comparative analysis reveals that the pulsed field mainly improves the average orientation angle of BNNSs but has limited effect on reducing their spacing; the DC field significantly reduces the inter-sheet distance but does not substantially enhance alignment; in contrast, the superimposed field simultaneously increases the average orientation angle and reduces spacing, achieving a more effective orientation and arrangement of BNNSs.

In summary, compared to the DC electric field, the superimposed electric field provides a larger electric force to BNNSs, promoting local orientation and increasing the average orientation angle of BNNSs. Compared to the pulse electric field, the superimposed electric field supplies a continuous and stable Coulomb force, facilitating mutual motion between BNNSs and enhancing global arrangement. From the perspective of forces acting on BNNSs in the electric field, it can be concluded that the superimposed electric field induces a higher degree of BNNS orientation and arrangement than either DC or pulse fields alone, forming more efficient thermal conduction pathways and thus improving the thermal conductivity of the composites.

### 3.4. Thermogravimetric Properties of Composites

**Figure 7:**
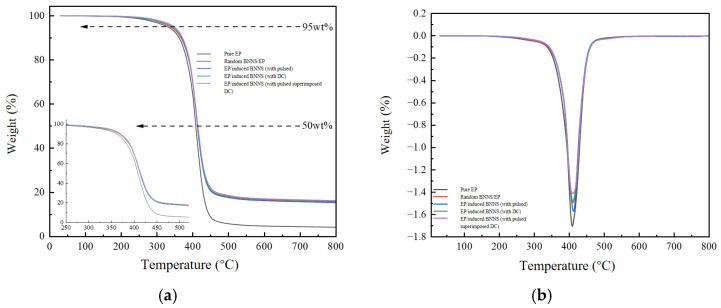
TGA and DTG curves of the composites. (**a**) TGA; (**b**) DTG.

Figure 7 shows the thermogravimetric analysis (TGA) curves and derivative thermogravimetry (DTG) curves of five materials. The thermal degradation behavior of the four composite materials exhibits a similar trend compared to the pure epoxy resin matrix, indicating that the incorporation of BNNSs into the epoxy resin does not alter the thermal decomposition mechanism of the matrix. Quantitative analysis of Figure 7 was performed to obtain the residual mass fraction at the final temperature of 800 °C, the temperatures corresponding to 5 wt% and 50 wt% weight loss for the five materials, as well as the maximum decomposition temperature (T_max_) corresponding to the peak of the DTG curve. These temperatures are denoted as T_5%_, T_50%_, and T_max_, respectively, and the data are summarized in Table 2.

From Table 2, it can be observed that after incorporating 10 wt% BNNSs into the matrix, the thermal decomposition temperatures of the composites have all increased. This phenomenon may be attributed to the addition of BNNSs, which reduces the amount of epoxy resin matrix in the sample. Additionally, due to the excellent intrinsic heat capacity properties of BNNSs, less epoxy resin matrix decomposes at the original characteristic temperatures, resulting in the need for higher temperatures to achieve the same mass loss.

### 3.5. Glass Transition Temperature of Composites

**Figure 8:**
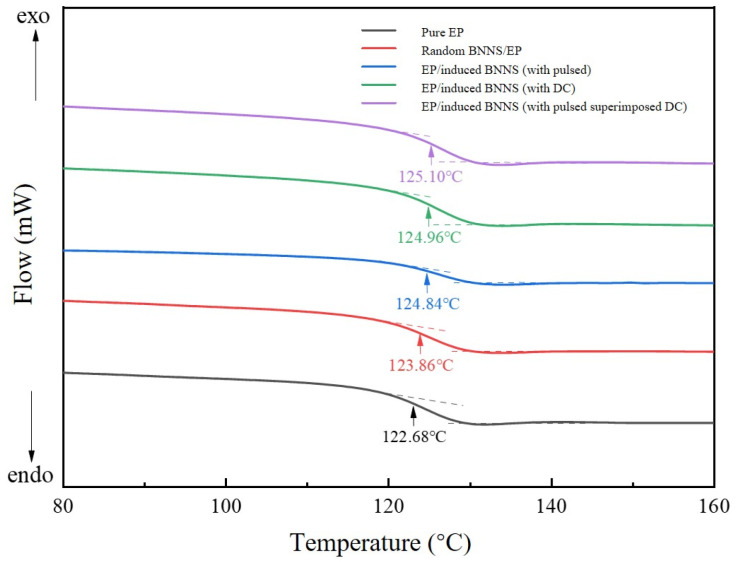
DSC curves of different samples.

From Figure 8, the glass transition temperatures (Tg) of the five samples can be obtained. Compared to pure epoxy resin, the other four composites exhibit higher glass transition temperatures. This is mainly because the surface groups of BNNSs interact with the epoxy molecules, restricting their mobility. Additionally, the incorporation of BNNSs creates a spatial steric hindrance effect, forming physical barriers within the matrix material that limit the rotation and movement of molecular chains [43]. As a result, more energy is required for the transition from the glassy state to the rubbery state. Therefore, after the addition of BNNSs, the glass transition temperature of the composites is slightly higher than that of pure epoxy resin. During the curing process, with the addition of the inducing electric field, BNNSs orient and connect with each other, moving relative to one another to form an overall “skeleton-like” rigid chain structure. This structure may further restrict the rotation and movement of molecular chains, so the molecular chains need to absorb more energy to overcome these constraints and enable their motion [30,44]. Therefore, compared to the random-type samples, the glass transition temperature of the electric field-induced samples is higher. Since the orientation and arrangement degree of BNNSs and molecular chains in the composite induced by the superimposed electric field is higher than that of the single induction-type composites, the glass transition temperature of the superimposed induction-type samples is further improved compared to single induction-type composites.

## 4. Conclusions

This paper used a pulsed superimposed DC electric field to induce the orientation arrangement of BNNSs in a thermosetting epoxy resin matrix, so as to construct efficient thermal conductive channels, improve the thermal conductivity of composites, and provide a new solution to the thermal aging problem caused by poor thermal conductivity of materials. The results show that the pulsed superimposed DC electric field can further construct efficient thermal conductive channels on the basis of a single electric field, improve the thermal conductivity of composites, and exert the synergistic effect of the pulsed electric field and DC electric field. The thermal conductivity of the composite prepared by superimposing 8 kV/mm pulsed electric field on 30 V/mm DC electric field is about 0.474 W/(m·K), which is 34.66% higher than that prepared by only an 8 kV/mm pulsed-induced field and 17.5% higher than the theoretical superposition value. The thermal conductivity of the composite prepared by superimposing a 4 kV/mm pulsed electric field on a 70 V/mm DC electric field increased to about 0.464 W/(m·K), which is 27.47% higher than that prepared by only a 70 V/mm DC-induced field and 12.4% higher than the theoretical superposition value. At the same time, the superimposed induced composites also have good thermal stability. Therefore, the composite prepared by inducing the orientation arrangement of BNNSs with a pulsed superimposed DC electric field can improve the thermal conductivity and thermal stability of the composite at the same time.

## Figures and Tables

**Table 1:** Comparison table of thermal conductivity of composite materials.

Filler Type and Content	Filler Type and Content	Thermal Conductivity *λ* (W/m·K)	Alignment Type	Cite
External DC electric field	No filler	0.091	Lattice vibration modulation (non-filler alignment)	[34]
Pulsed square-wave electric field	h-BN, 10 wt%	0.453	In-plane alignment	[22]
AC/DC electric field	h-BN, 15 vol%	1.56	Linear densely packed bundles (LDPBNs)	[21]
DC electric field	h-BN, 10 wt%	0.544	Out-of-plane vertical alignment	[35]
AC/DC electric field	BN: 25 wt%; SiC: 5 wt%	0.3425	Hybrid filler co-alignment	[36]
Pulsed Superimposed DC Electric Field	BN:10%	0.474	Local and global alignment	This paper

**Table 2:** Characteristic thermogravimetric analysis data of different samples.

Samples	T_5%_/°C	T_50%_/°C	T_max_/°C	Weight Loss Rate (800 °C)
Pure EP	329.2	408.5	408.8	94.86%
Random BNNS/EP	336.5	413.2	410.2	84.40%
EP/induced BNNS (with pulsed)	342.7	413.3	411.5	84.77%
EP/induced BNNS (with DC)	342.8	414.0	410.8	84.10%
EP/induced BNNS (with pulsed superimposed DC)	347.7	414.3	410.0	83.79%

## Data Availability

Data is contained within the article: the original contributions presented in this study are included in the article. Further inquiries can be directed to the corresponding author.

## Supplementary Materials

The following supporting information can be downloaded at: https://www.mdpi.com/article/10.3390/mi16101126/s1, Figure S1: shows the SEM and EDS image of the sample.
