# Supplementary material for: The Influence of Pulsed Superimposed DC Electric Field Synergistically Inducing Orientation Arrangement of BNNSs on Thermal Properties of Epoxy Composites
Source: Micromachines (Basel). 2025 Sep 30;16(10):1126. doi: 10.3390/mi16101126 (PMC12566423; doi:10.3390/mi16101126)
Supplement: Supplementary file 1 [file micromachines-16-01126-s001.zip › micromachines-3862143-supplementary.pdf]

For research article

Supporting Informations

We have added SEM-EDS analysis as shown in Figure S1. The EDS mapping of the B element effectively reflects its distribution, which appears relatively dispersed, mainly due to magnetic stirring and ultrasonic treatment during the preparation process. However, combined with the SEM images, locating individual BNNS remains challenging. After reviewing numerous references and analyzing the principles of EDX, it was found that EDS is primarily used to reveal the elemental distribution of fillers forming chain-like structures within the matrix, enabling clear differentiation between fillers and matrix and the identification of chain formation. Nevertheless, identifying individual BNNS is difficult because EDS detection involves micron-level errors, and its detection depth (0.1–5 μm) may interfere with SEM surface analysis. Therefore, we did not employ EDS detection in this study.

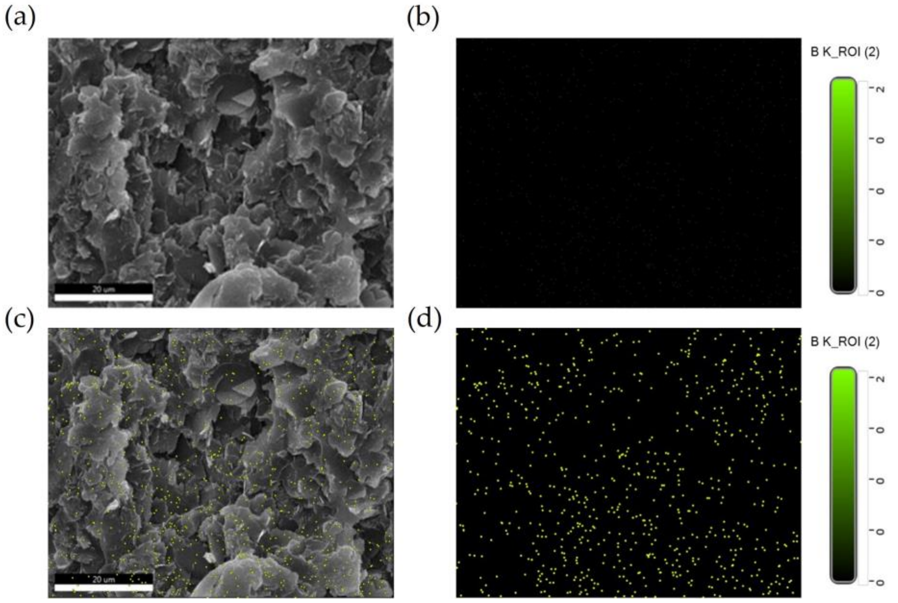

Figure S1. shows the SEM and EDS image of the sample. (a) is the SEM of the sample, (b) presents the EDS mapping of the B element (the N element is not displayed, considering its presence in the epoxy resin curing agent), (c) is integrates the SEM and EDS images, (d) shows the brightness-enhanced version of (b).
